# Supplementary material for: Patterns of Intron Gain and Loss in Fungi
Source: PLoS Biol. 2004 Nov 30;2(12):e422. doi: 10.1371/journal.pbio.0020422 (PMC532390; doi:10.1371/journal.pbio.0020422)
Supplement: Table S1 — Also available at http://genes.mit.edu/NielsenEtAl/. (4.3 MB ZIP). [file pbio.0020422.st001.zip › NielsenEtAl/html/406.html]

AN0381.1.NCU02207.1.MG01979.1.FG05778.1


```
 CLUSTAL W (1.82) Multiple Sequence Alignments - Introns Inserted


Sequence 1: MG01979.1	530 aa
Sequence 2: FG05778.1	528 aa
Sequence 3: NCU02207.1	539 aa
Sequence 4: AN0381.1	531 aa
Alignment Length: 540 aa
Number Identitical Residues: 426 aa
Alignment Score (without introns) 16548


MG01979.1 	---------M0S~SFS-PTQIFEEGTTEEKGENARLSAFVGAIAVGDLVKSTLGPKGMDK
NCU02207.1	MMAAGSGLSI0A~SFS-PTQIFEEGTTEEKGENARLSAFVGAIAVGDLVKSTLGPKGMDK
FG05778.1 	----------~M0SFQ-PTQIFEEGTTEEKGENARLSAFVGAIAVGDLVKSTLGPKGMDK
AN0381.1  	---------M0A~SFANPTQIFADDVIEEKGENARLSAFVGAIAVGDLVKSTLGPKGMDK
          	             ** .***** :.. *********************************

MG01979.1 	ILQSA2STGEIMVTNDGATILKSIALDNAAAKVLVNISKVQDDEVGDGTTSVSVLAAELL
NCU02207.1	ILQSA2STGEILVTNDGATILKSIALDNAAAKVLVNISKVQDDEVGDGTTSVAVLAAELL
FG05778.1 	ILQSA2STAEIMVTNDGATILKSIALDNAAAKVLVNISKVQDDEVGDGTTSVAVLAAELL
AN0381.1  	ILQSA2STGEILVTNDGATILKAIALDNAAAKVLVNISKVQDDEVGDGTTSVTVLAAELL
          	***** **.**:**********:*****************************:*******

MG01979.1 	REAEILVGKKIHPQTIIEGYRIASRAALEALEQSAVDHSKNPEAFKKDLLAIARTTLSSK
NCU02207.1	REAEKLVDKKIHPQTIIEGYRIASQAALKALEDSAVDHSSNPEAFRKDLVAIARTTLSSK
FG05778.1 	REAEKLVDKKIHPQTIIEGYRIASHAALKALEGSAVDHSKNPEAFRQDLLAIARTTLSSK
AN0381.1  	REAEKLVNRKIHPQTIIEGYRIASRAALDALDKVAVDRSDDMEKFRKDLHSIARTTLSSK
          	**** **.:***************:***.**:  ***:*.: * *::** :*********

MG01979.1 	VLAQDRDHFSRLACEAVLRLKDSSDLSHIQIIKKAGGKLSDSFLDEGFILDKKIGVNQPK
NCU02207.1	ILAQDRDHFAELATEAILRLKGSADLSHIQIIKKSGGKLSESYLDDGFILDKKIGVNQPK
FG05778.1 	VLAQDRNQFAELAVDAVLRLR-SSDLNHIQIIKKAGGKLSDSYLDEGFILDKKIGVNQPK
AN0381.1  	VLAQDRDQFAALACDAVLRLRGSTDLSHIQIIKKAGGKLSDSYLDEGFILDKKIGVNQPK
          	:*****::*: ** :*:***:.*:**.*******:*****:*:**:**************

MG01979.1 	RLENAKILVANTSMDTDKVKIFGARMKVGSTSKLAELEKAEKDKMKAKVDKIKAHGINCF
NCU02207.1	RLEKAKILIANTSMDTDKVKIFGARVKVNSTSKLAELEKAEKEKMKAKVEKIKAHGINCF
FG05778.1 	RLEKAKILVANTSMDTDKVKIFGARVKVGSTSKLAELEKAEKDKMKAKVEKIKAHGINCF
AN0381.1  	RLENAKILVANTAMDTDKVKIFGARVKVESTGKLAELEKAEREKMKAKVERIKAHGINCF
          	***:****:***:************:** **.*********::******::*********

MG01979.1 	INRQLIYNWPEQLFTDAGIMSIEHADFDGIERLALVTGGEITSTFDHPDQVKLGHCDLIE
NCU02207.1	INRQLIYNWPEQLFTDAGIMSIEHADFDGIERLALVTGGEIASTFDHPEQVKLGYADLIE
FG05778.1 	INRQLIYNWPEQLFTDAGIMSIEHADFDGIERLALVTGGEIASTFDHPEQVKLGHCDTIE
AN0381.1  	VNRQLIYNWPEQLFTEAGIMSIEHADFDGIERLALVTGGEIASTFDHPDQVKLGSCDLIE
          	:**************:*************************:******:***** .* **

MG01979.1 	EIIIGEDTLIKFSGVAAGEACTIVLRGATEQLLDEAERSLHDALAVLSQTVKEPRTTLGG
NCU02207.1	EIIIGEDTLIRFSGTAAGQACTVVLRGATDQLLDEAERSLHDALAVLSQTVKESRTTLGG
FG05778.1 	EVIIGEDTLIKFSGVSGGEACTIVLRGATEQLLDEAERSLHDALAVLSQTVKEPRTTLGG
AN0381.1  	EVIIGEDTLIKFSGVAAGQACTIVLRGATEQLLDEAERSLHDALAVLSQTVKDPRVTLGG
          	*:********:***.:.*:***:******:**********************:.*.****

MG01979.1 	GCAEMVMAKAVEGAATRVEGKRQMAVASFATALRQLPTILADNAGLDSGELVARLRKAIY
NCU02207.1	GCAEMVMAKAVEGAATRVEGKKQTAVSAFATALRQLPTILADNAGLDSGDLVARLRKAIY
FG05778.1 	GCAEMLMAKAVEGAATRVEGKRQLAVQSFAIALRQLPTILADNAGLDSGDLVARLRKALY
AN0381.1  	GCAEMVMSKAVEQAAQNTTGKKQLAVDSFAHALKQLPTILADNAGLDSSDLVTRLRTAIN
          	*****:*:**** ** .. **:* ** :** **:**************.:**:***.*: 

MG01979.1 	DGMTTYGLDLMTPGGGIADMRDIGVIESYKLKKAVVSSASEAAE0LLLRVDDIIRAAPRR
NCU02207.1	DGLTTYGLDLMTPGGGIADMRDLGVIESYKLKKAVVSSASEAAE0LLLRVDDIIRAAPRR
FG05778.1 	DGLTTYGLDLMTPGGGITDMRDLGVIESYKLKKAVVSSASEAAE0LLLRVDDIIRAAPRR
AN0381.1  	NGMTSSGLDLLTPGGGIADMRELGVVESYKLKKAVVSSASEAAE0LLLRVDNIIRAAPRR
          	:*:*: ****:******:***::**:****************** ******:********

MG01979.1 	RERQ~
NCU02207.1	RERH~
FG05778.1 	RERI2
AN0381.1  	RDRM~
          	*:*
```
